# Bone Marrow MicroRNA-335 Level Predicts the Chemotherapy Response and Prognosis of Adult Acute Myeloid Leukemia

**DOI:** 10.1097/MD.0000000000000986

**Published:** 2015-08-21

**Authors:** Li Yingchun, Zhang Rong, Yao Kun, Yang Ying, Liu Zhuogang

**Affiliations:** From the Department of Hematology, Shengjing Hospital, China Medical University, Shenyang, China.

## Abstract

The aim of this study was to investigate the role of microRNA-335 (miR-335) in determining the treatment response and prognosis in adult acute myeloid leukemia (AML) patients receiving the cytarabine (Ara-C)-based chemotherapy.

A total of 204 adult AML patients were collected. The miR-335 levels in serum and bone marrow samples from these patients were determined. All patients received Ara-C-based standard induction chemotherapy regimens. The treatment response to Ara-C-based chemotherapy was evaluated. All patients were followed for prognostic analyses.

The levels of miR-335 in bone marrow and serum samples from adult AML patients achieving complete response were significantly higher than those without. The serum miR-335 level was not associated with the chemotherapy response and prognosis in these AML patients. In contrast, high bone marrow miR-335 level was significantly associated with a poor treatment response and also predicted a worse prognosis indicated by the relapse-free survival and overall survival periods in adult AML patients receiving Ara-C-based chemotherapy.

Our finding suggests that bone marrow miR-335 level may be used as a marker to predict the chemotherapy response and prognosis in adult AML patients.

## INTRODUCTION

Acute myeloid leukemia (AML) is a malignant disorder characterized by the unlimited proliferation of granulocytic, monocytic, and megakaryocytic cells in blood.^1^ Current therapy for AML consists of cytotoxic chemotherapy followed by consolidation chemotherapy or stem cell transplantation.^2,3^ However, the prognosis of AML patients remains very poor. Although some cytogenetic features are reported to be associated with the clinical outcome,^4–7^ the prognosis of AML patients cannot yet be predicted with adequate accuracy. Thus, it is of great clinical significance to identify novel and effective markers, which can predict the chemotherapy response and prognosis of AML patients.

MicroRNAs (miRNAs) are a class of small, noncoding RNA molecules (usually 18–25 nucleotides in length), which have been identified as important regulators of approximately 30% of all protein-coding RNAs.^8^ MiRNAs play critical roles in multiple biological processes, including development, embryogenesis, cellular proliferation and differentiation, organogenesis, and apoptosis. Recently, several miRNAs have been documented to play important roles in tumorgenesis and metastasis. MicroRNA-335 (miR-335) is one of these tumor-related miRNAs. MiR-335 inhibits migration of breast cancer cells through targeting the oncoprotein c-Met.^9^ Methylation-associated silencing of miR-335 contributes tumor cell invasion and migration in gastric cancer.^10^ Upregulation of miR-335 predicts a favorable prognosis in esophageal squamous cell carcinoma.^11^

In blood tumor, a previous study reported that miR-335 levels in bone marrow samples from pediatric AML patients were significantly higher than those from normal controls, and this aberrant expression of miR-335 is associated with tumor progression and prognosis.^12^ However, it should be noticed that some miRNAs have different expression pattern between pediatric and adult patients.^13^ Thus, the role of miR-335 in adult AML still needs to be identified. In this study, we investigated the roles of serum and bone marrow miR-335 levels in determining the treatment response and clinical outcome in adult AML patients receiving cytarabine (Ara-C)-based standard chemotherapy.

## METHODS

### Ethics Statement

This study was approved by the Institute's Review Board of China Medical University and conducted according to the Helsinki declaration. The written consent was obtained from all patients and healthy controls.

### Enrollment

A total of 204 adult patients diagnosed with AML were collected from our hospital. The diagnosis of AML was made according to a morphologic assessment of the Wright–Giemsa-stained smears of the bone marrow aspirates along with special stains and immunophenotyping by flow cytometry. Patients with extramedullary disease and those who received allogeneic stem cell transplantation were excluded. The acute promyelocytic leukemia (M3) was also excluded because of different treatment regimens. All participants were ethnically of Chinese Han.

### Evaluation of Chemotherapy Regimens and Therapeutic Response

All adult AML patients enrolled in this study underwent the Ara-C-based standard induction chemotherapy. Of all 204 patients, there were 65 who received daunorubicin 45 mg × m-2 × d-1 for 1 to 3 days and Ara-C 100 mg × m-2 × d-1 for 1 to 7 days (DA induction chemotherapy); 97 received homoharringtonine (HHT) 3 to 4 mg × m-2 × d-1 for 5 to 7 days and Ara-C 100 mg × m-2 × d-1 for 1 to 7 days (HA induction chemotherapy); and 42 received mitoxantrone 4 mg × m-2 × d-1 for 1 to 5 days and Ara-C 100 mg × m-2 × d-1 for 1 to 7 days (MA induction chemotherapy).^14^ For outcome analyses, we evaluated if patients reached complete response (CR), which was defined as follows previously. Patients with other treatment response, including partial remission, nonremission, and early death, were assigned as non-CR group.^14^ For long-term prognosis analyses, we adopted the relapse-free survival (RFS) and overall survival (OS) in this study. RFS was defined as the interval between the achievement of complete remission and the time of the hematological relapse or the last follow-up. OS was defined as the interval between the moment of diagnosis and death or the last follow-up.^15^

### Real-Time Quantitative RT-PCR for miRNA

The miR-335 expression levels in bone marrow mononuclear cells and serum were detected by the real-time quantitative Reverse Transcription-Polymerase Chain Reaction (RT-PCR) as described elsewhere. The primer sequences used for RT-PCR were as follows—miR-335: forward 5′-GCG GTC AAG AGC AAT AAC GAA-3′, reverse 5′-GTG CAG GGT CCG AGG TAT TC-3′; RNU6B: forward 5′-CGC TTC GGC AGC ACA TAT AC-3′, reverse 5′-TTC ACG AAT TTG CGT GTC AT-3′. Relative quantification of target miRNA expression was evaluated using the comparative cycle threshold method. The raw data were presented as the relative quantity of target miRNA, normalized with respect to RNU6B. Each sample was tested in triplicate.^16^

### Statistical Analysis

The comparison of the characteristics of AML patients achieving CR and those did not achieved CR were analyzed by using Student *t* test (age, white blood cell count, hemoglobin, platelet, serum, and bone marrow miR-335 levels) or χ^2^ test (sex, smoker, leukemia type). The Kaplan–Meier survival curves were used to determine any relationship between the miR-335 levels and the status of the patients with respect to RFS or OS. The univariate and multivariate Cox analyses were used to assess the independent prognostic values of serum and bone marrow miR-335 levels with adjustment of age, sex, smoking status, white blood cell count, hemoglobin, platelet counts, and leukemic types. The hazard ratio (HR) and its 95% confidence interval (95%CI) were calculated. The software of SPSS version 16.0 for Windows (SPSS Inc, IL) was used for statistical analysis. Differences were considered statistically significant when *P* was <0.05.

## RESULTS

Among these patients with AML, 4 patients were diagnosed with AML without maturation (M1); 45 patients with maturation (M2); 70 patients with acute myelomonocytic leukemia (M4); 65 patients with acute monocytic leukemia (M5); 12 patients with erythroleukemia (M6); and 8 patients with acute megakaryoblastic leukemia (M7). There are 133 patients who had achieved CR (CR group), whereas the rest of 71 were assigned into non-CR group. The characteristics of these 2 groups are shown in Table 1. There was no significant difference in age, sex, and leukemia type between CR and non-CR groups (both *P* > 0.05). The patients in the CR group had a higher smokers rate than those from non-CR group (*P* = 0.001). The miR-335 levels in the bone marrow and serum samples from CR patients were significantly higher than non-CR patients (both *P* < 0.001).

**TABLE 1:**
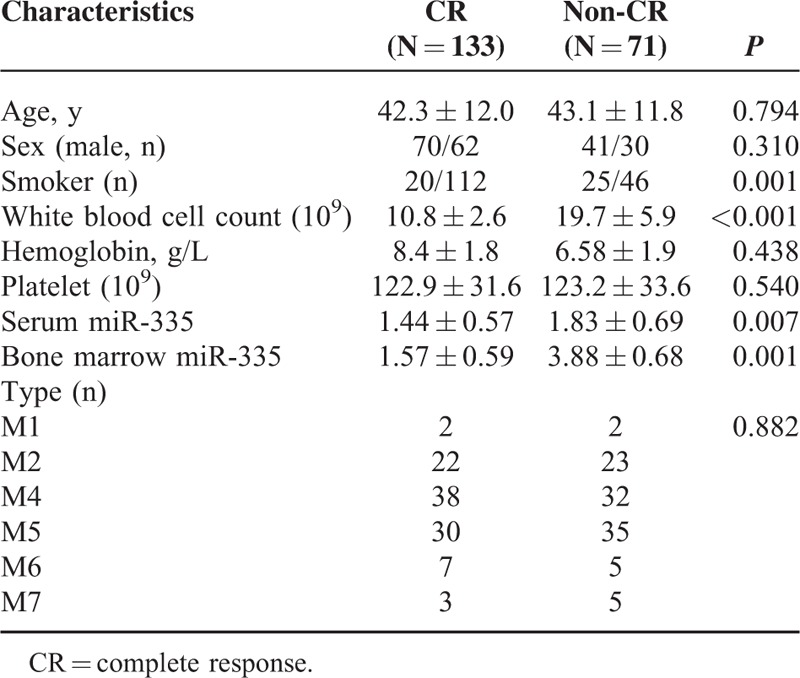
The Characteristics From Patients and Controls

We next investigate the association of miR-335 expression levels in serum and bone marrow samples with chemotherapy response. Using the mean value of serum and bone marrow miR-335 as cutoff values, all patients were divided into low miR-335 and high miR-335 expression groups. As shown in Table 2, the high bone marrow miR-335 group had more nonresponders than low miR-335 group. In contrast, there was no difference in response status between patents with high and low serum miR-335. Multiple logistic regression analyses revealed that high miR-335 level in bone marrow had an increased possibility of having poor response to chemotherapy (odds ratio = 2.63, 95% CI: 1.45–4.74, *P* < 0.001) with adjustment of age, sex, smoking status, white blood cell count, hemoglobin, and platelet counts. The serum miR-335 expression level, however, was not associated with the chemotherapy response in these patients (odds ratio = 1.32, 95% CI: 0.77–2.44, *P* = 0.178).

**TABLE 2:**
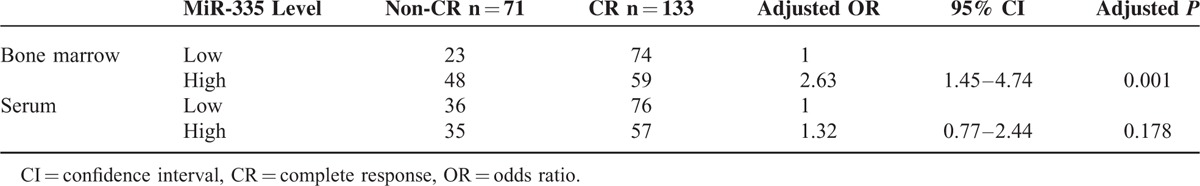
The Association of miR-335 Expression Levels in Serum and Bone Marrow With Chemotherapy Response

**FIGURE 1:**
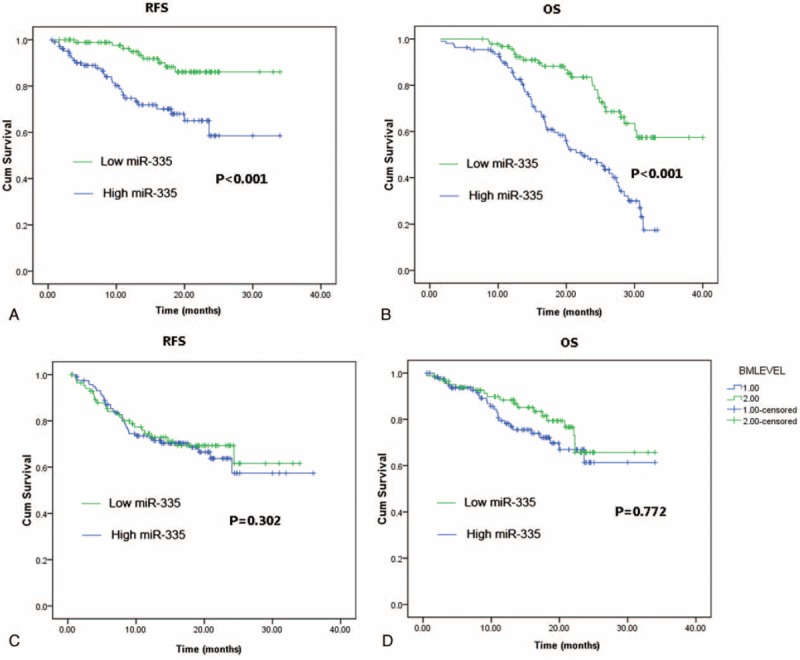
(A) and (B) show that AML patient with high bone marrow miR-335 level had shorter RFS and OS than those with low miR-335 expression (both *P* < 0.001 by log-rank analyses). In contrast, the serum miR-335 expression did not predict the prognosis of AML patients (both *P* > 0.05 by log rank analyses—C and D). AML = acute myeloid leukemia, RFS = relapse-free survival, OS = overall survival.

All AML patients were followed for prognosis analysis. The median follow-up duration was 26.5 months ranged from 1.2 to 48 months. Figure 1 shows the Kaplan–Meier curves for RFS and OS stratified by the miR-335 level status (high and low) in serum and bone marrow samples in these patients. Our data revealed that high miR-335 expression in bone marrow presented a poorer outcome indicated by shorter RFS and OS than low miR-335 expression (both *P* < 0.001 by log-rank analyses; Figure 1 A and B). However, the serum miR-335 expression was not associated with the prognosis of AML patients (both *P* > 0.05 by log-rank analyses; Figure 1 C and D).

We further conducted the univariate and multivariate Cox analysis to confirm the prognostic factor of AML patients with adjustment of age, sex, smoking status, white blood cell count, hemoglobin, platelet counts, and leukemic types. We identified that chemotherapy response and miR-335 expression as factors related to AMP prognosis by univariate Cox analysis. We next performed the Cox proportional hazards multivariate analysis of the univariate predictors to determine the independent prognostic factors for RFS and OS. Both chemotherapy response and miR-335 expression were identified as independent prognostic factors for RFS and OS (Table 3).

**TABLE 3:**
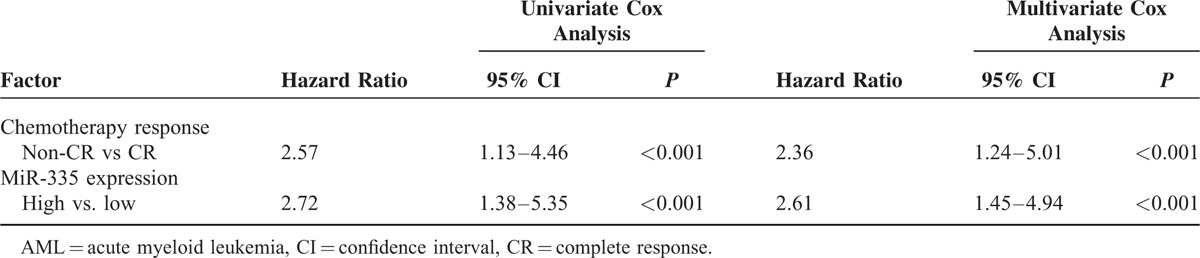
The Prognostic Factor of AML by Univariate and Multivariate Cox Analysis

## DISCUSSION

In the current study, we showed that the expression levels of miR-335 in bone marrow and serum samples from adult patients with AML (except M3) were significantly associated with the Ara-C-based chemotherapy response and clinical outcome after treatment. High miR-335 expresser status was correlated with poor treatment response and worse prognosis in these patients. This finding is of clinical importance because there is no reliable molecular marker to predict the chemotherapy response in adult AML patients.

Deregulation of miRNA has been implicated in many types of solid tumors as well as in hematologic malignancies. In AML, they have been shown to influence hematopoietic mechanisms of cell proliferation and differentiation, to link with the genetic heterogeneity, and to impact on treatment response.^17^ Both miRNA signatures and a single miRNA have been demonstrated to provide prognostic information for risk assessment in AML.^18–20^ Furthermore, data from the first clinical phase 2 study revealed that the miRNA-targeted drug—antimiR-122—was safe and effective, which indicates that miRNA-based therapeutics could become a reality in clinical medicine.^21^

MiR-335 is an intragenic miRNA residing in the second intron of the mesoderm-specific transcript *Mest*. During normal development, miR-335 targets key endoderm transcription factors downstream of Nodal/TFGâ and Wnt/â-catenin pathways.^22^ Tome et al^23^ demonstrated that miR-335 is part of a common regulatory pathway for cell proliferation, migration, and differentiation in human mesenchymal stem cells. In addition, differential expression of miR-335 has been linked to several types of cancers; however, its function in tumor initiation and progression is controversial. In breast cancer, overexpression of miR-335 resulted in an increase in tumor suppressor gene *BRCA1*, leading to decreased cell viability and increased apoptosis.^24^ In small cell lung cancer, loss of miR-335 promoted metastatic skeletal lesions via deregulation of IGF-IR and RANKL pathways.^25^ On the contrary, the potential oncogenic role of miR-335 was identified by showing its elevated expression in malignant astrocytomas, gastric cancer recurrence samples, and pediatric AML.^12,13,26–29^

The result of our study provides evidence of a close relationship between bone marrow miR-335 expression levels and clinical outcome in adult AML patients. Although higher pretreatment levels of miR-335 in serum and bone marrow were observed in AML patients, increased levels of miR-335 only in the bone marrow were associated with lower odds of achieving CR and shorter RFS and OS. Moreover, we found that miR-335 expression levels, in addition to chemotherapy response, constitute an independent prognostic factor in patients with AML even when other validated clinical prognosticators were considered in multivariable models.

To our knowledge, this is the first clinical study reporting that high bone marrow miR-335 levels conferred shorter event-free survival and OS in adult AML patients. Interestingly, the oncogenic role of miR-335 in pediatric AML has been shown by 2 independent groups. One group found that miR-335 in bone marrow samples was upregulated in pediatric M1 patients compared with the normal control.^13,30^ The other group observed that serum miR-335 was markedly and consistently increased in childhood AML and may serve as a marker for monitoring disease progression in these patients.^12,29^ Thus, in line with the observations made in pediatric patients, our results further suggest the leukemogenic function of miR-335 in adult AML patients.

Several limitations should be addressed in our study. First, further studies that use a larger number of samples from different countries are needed to confirm the prognostic significance of miR-335 in AML patients. Second, we did not assess the possible chromosome aberration and genetic alteration in these patients, which might become the hidden confounding factors. Third, it would be interesting for future studies to look for patterns in gene expression profiles associated with miR-335 expression.

In summary, we report that high expression levels of miR-335 bestow a relatively unfavorable clinical profile in adult patients with AML; and the expression of miR-335 in bone marrow samples is independently associated with clinical outcome in these patients. Accordingly, measurement of miR-335 expression may provide additional prognostic information for a better risk stratification of patients with AML. Moreover, the clinical development of compounds capable of decreasing miRNA expression in vivo may provide novel therapeutic options for patients with high miR-335 expression levels.
